# Has variation in length of stay in acute hospitals decreased? Analysing trends in the variation in LOS between and within Dutch hospitals

**DOI:** 10.1186/s12913-015-1087-6

**Published:** 2015-09-30

**Authors:** Aart R. van de Vijsel, Richard Heijink, Maarten Schipper

**Affiliations:** National Institute for Public Health and the Environment, Richard Heijink, P.O. Box 1, 3720 BA Bilthoven, The Netherlands

**Keywords:** Length of stay, Hospital efficiency, Practice variation, Multilevel analysis

## Abstract

**Background:**

We aimed to get better insight into the development of the variation in length of stay (LOS) between and within hospitals over time, in order to assess the room for efficiency improvement in hospital care.

**Methods:**

Using Dutch national individual patient-level hospital admission data, we studied LOS for patients in nine groups of diagnoses and procedures between 1995 and 2010. We fitted linear mixed effects models to the log-transformed LOS to disentangle within and between hospital variation and to evaluate trends, adjusted for case-mix.

**Results:**

We found substantial differences between diagnoses and procedures in LOS variation and development over time, supporting our disease-specific approach. For none of the diagnoses, relative variance decreased on the log scale, suggesting room for further LOS reduction. Except for two procedures in the same specialty, LOS of individual hospitals did not correlate between diagnoses/procedures, indicating the absence of a hospital wide policy. We found within-hospital variance to be many times greater than between-hospital variance. This resulted in overlapping confidence intervals across most hospitals for individual hospitals’ performances in terms of LOS.

**Conclusions:**

The results suggest room for efficiency improvement implying lower costs per patient treated. It further implies a possibility to raise the number of patients treated using the same capacity or to downsize the capacity. Furthermore, policymakers and health care purchasers should take into account statistical uncertainty when benchmarking LOS between hospitals and identifying inefficient hospitals.

**Electronic supplementary material:**

The online version of this article (doi:10.1186/s12913-015-1087-6) contains supplementary material, which is available to authorized users.

## Background

In-patient length of stay (LOS) has been widely used as an indicator of hospital performance, predominantly as an indicator of the efficiency of the hospital delivery process [1–7]. Besides, LOS may be used as quality indicator, though the attribution of causal association is problematic [8]. LOS is affected by supply and demand factors. Therefore, after correction for demand factors, differences in LOS can be attributed to supply factors like doctors’ treatment choices and hospital management. Hence, these differences may indicate underuse or overuse including supplier-induced demand [9].

The average LOS (ALOS) has declined for decades and continues to drop in the industrialised world, both for all hospital care and specific diagnoses [10, 11]. This phenomenon can be explained by the introduction of new treatment modalities, such as minimal invasive procedures and fast track programmes for major surgery [12–14], and the streamlining and rationalisation of care processes through clinical pathways (CP’s) [15, 16].^1^ Besides, substantial variation in LOS has been observed between countries [6, 11], regions and hospitals, for individual conditions and procedures (e.g. [1, 2, 16–20]), individual medical specialties and all hospital care [3, 4]. These variations were found even after controlling for various patient characteristics (case-mix). Several studies explained part of the variation in case-mix adjusted LOS by the accessibility of hospitals, organisational factors, or the availability of formal and informal aftercare [7, 19, 21, 22].

Only a few studies investigated trends in LOS variation,^2^ even though insight into the development of LOS variation within and between hospitals can be important from a scientific and a policy perspective. First, reducing unwarranted practice variation, also in terms of LOS, is an important aim of CPs [14, 23]. Second, declining and stabilising variation may indicate that there is little room left for further efficiency improvement, whereas increasing variation may indicate the contrary. One study reported a decrease of LOS variation for inguinal hernia repair after the implementation of a CP [23]. Two studies investigated trends in geographic variation in LOS in the US: for cerebrovascular diseases between 1963 and 1991 [16] and for uncomplicated deliveries between 1988 and 1995 [24]. In both studies, the US was divided into four regions, which showed similar declines in LOS. The relevance of the variation between areas is limited because of their large size. Summarizing, we conclude that there is little insight into changes in the variation between and within hospitals in LOS over time. Even the more general literature on medical practice variation (hospital service utilization) includes only a few studies examining changes over time. These studies mainly concentrated on admission and procedure rates [25]. Westert et al. [25] and Weinstein et al. [26] found a declining (Westert) and a stable or only slightly declining (Weinstein) variation between regions in admission rates for high-volume procedures in the Netherlands and US respectively. Both studies found a constant ranking of regions over time.

### Aim and research questions

The aim of this paper was to provide more insight into the development of variation in LOS within and between hospitals for multiple diagnoses and procedures. More specifically, we intended to establish whether the reduction of the ALOS was accompanied by a reduction of the variation in LOS within and between hospitals?

In addition, we focused on the following two questions. First, do hospitals with a relatively high standardised LOS at the beginning of the observed period keep this position over time? In other words, is the ranking of hospitals in terms of LOS constant over time? Second, is the streamlining of care processes a hospital wide policy? Do hospitals with a relatively short LOS for one diagnosis or procedure also have a short LOS for the other diagnoses or procedures?. For a better understanding of the results, we first provide some background information on the Dutch hospital sector. Following that, we will describe the analysis and results.

## Dutch hospital care

We focussed on Dutch hospital care where ALOS for in-patients dropped from 13.1 days in 1981 to 9.5 days in 1995 and 5.6 days in 2010.^3^ Cross-sectional studies found marked differences in LOS between Dutch hospitals, for all conditions and for separate specialties and diagnoses [3, 4]. A reduction of the variation in LOS *within* Dutch hospitals was expected since the beginning of this century, because of the streamlining and rationalisation of care processes by applying instruments like CP’s. These trends were influenced by various factors, such as the development of evidence based medicine, pressure to reduce waiting times (access time) and possibly changes in the manner of reimbursement of hospitals. The Dutch hospital reimbursement system was radically changed in 2001. Fixed budgets were replaced by budgets that were to a large extent volume based and open ended. Moreover, the lump sum for doctors’ fees was turned into payment based on realised hospital output. This resulted in a steep increase in the number of admissions [27]. Given the limitations on hospital capacities, this provided an incentive to reduce LOS further through the streamlining of care processes. Finally, since 2005, a reimbursement system that is more or less similar to the widely used Diagnosis Related Group (DRG) system has been used,^4^ in combination with a system of regulated competition and freely negotiable prices. The latter could lend pressure to the implementation of new treatment modes in order to streamline care processes and reduce costs.

As to the development of the variation *between* hospitals, we expected decreasing variation because of a more rapid diffusion of information on medical practice across providers, more publicity about new treatment modes and public pressure to adopt them. Between-hospital variation is also affected by differences in capacity constraints. Government policy up to the beginning of this century has led to a reduction in, and a more equal distribution of, the number of available beds per population served. The latter also contributes to diminishing variation in LOS between hospitals.

## Methods

### Setting and approach

We studied LOS in general and university hospitals in the Netherlands over the period 1995–2010 for a fixed group of hospitals. In order to exclude the effect of hospital mergers on changes in between-hospital variation, we bundled the data of the constituting hospitals for the years preceding the merger. In contrast to most previous studies, we analysed variation between (and within) hospitals instead of regions. We deemed this perspective more relevant, because: a) hospital management and doctors are the main decision makers in the hospital care delivery process, and b) developments between hospitals may differ from developments within hospitals. Furthermore, a disease and procedure specific perspective was used, because the extent to which care processes lend themselves to standardisation will differ across diagnoses and procedures. As a result, the variation within and between hospitals was expected to evolve differently for the various diagnoses and procedures. For example, hip and knee replacements are well-established procedures with a high volume belonging to the main medical areas where clinical pathways are used. Therefore, we expect relatively little LOS variation for these procedures.

### Selection of diagnoses and procedures

We selected diagnoses and procedures in such a way that they include: emergency and elective admissions; diagnoses that differ with regard to the degree of predictability and hence variability of the care process; conditions with fixed and those with freely negotiable product prices. Furthermore, for all selected diagnoses and procedures, care is mostly provided in an in-patient setting in the Netherlands (so the results were not influenced by differences between hospitals in the extent to which patients are treated in day care or outpatient care) and the volume was sufficient for reliable statistical analysis. The selected diagnoses and procedures are presented in Table 1, together with the ICD-9 codes for the diagnoses and the abbreviations used in this paper. The medical procedures are based on the Dutch classification of procedures.

**Table 1:** Selected diagnoses and medical procedures

Primary diagnosis or medical procedure	ICD-9 code^a^	Abbreviation
Femur fracture	820	FEMUR
Cerebrovascular accident	430–434, 436, 437	CVA
Chronic heart failure	428	CHF
Acute Myocardial Infarction plus Unstable Angina	410 + 411.1	AMIplus
Pneumonia	480–486	PNEU
Total hip replacement	-	HIP
Total knee replacement	-	KNEE
Partial resection of the colon (colectomy)	-	RESCOL
Cholecystectomy (bile stones)	-	CHOL

^a^The medical procedures hip replacement, total knee replacement, partial resection of the colon and cholecystectomy were selected using the Dutch classification of procedures

### Data

We used anonymised individual patient-level hospital admission data from the Dutch Hospital Discharge Register (DHDR) held by Dutch Hospital Data. The DHDR contains national administrative data from inpatient and day care admissions including several hospital, patient, and admission characteristics. The dataset contained records of patients that could be linked with the National Population Register (NPR) and were uniquely identifiable. The primary linkage key was based on date of birth, gender and postal code. On average, 87.5 % of the records could be uniquely linked for the year 2001 and the quality of the linkage was good [28]. In 1995, almost all hospitals participated in the DHDR. However, after 2005, participation declined, resulting in a coverage of 87 % of all admissions in 2009. Furthermore, from 2005 onwards, some hospitals ceased to register medical procedures. We excluded hospitals that did not participate in the DHDR or did not register procedures for more than 2 years in a row or in both 2009 and 2010. We could not include specialised hospitals and specialised treatment centres in our research because of lack of data. Out of the 92 general and university hospitals in 2010, 61 were included in the dataset. The admissions of the included hospitals that could be uniquely linked to the NPR represented approximately 75 % of the total number of in-patient admissions. Finally, we excluded those patients who died in hospital. Information on socioeconomic characteristics was obtained from a dataset of the Netherlands Institute for social research (SCP)^5^ containing the average socioeconomic status (SES) of the patient’s neighbourhood. These data were linked to the DHDR by (four-digit) postal code.

### Case-mix variables

In order to adjust for the impact of case-mix on the variation in LOS within and between hospitals, we included several case-mix variables. We adjusted for age using six classes: 0–25; 25–40; 40–55; 55–70; 70–85; 85+. For AMIplus (for definitions of the abbreviations: see Table 1), we adjusted for the type of medical procedure, because the diagnosis may not be sufficiently specific and the treatment policy of the hospital is a given for our analysis. Likewise, we adjusted RESCOL and CHOL for the way the procedure was carried out, i.e. with an open procedure or laparoscopic. To adjust for co-morbidity we used the Charlson index with 17 diagnosis groups based on ICD-codes [29, 30]. We re-calibrated the weights to the local and current context, because the weights of the original index are 25 years old and derived in the US [31]. To do so, we used weights developed for use in the actual Dutch situation by “De Praktijk Index”^6^ in collaboration with Dr Foster Intelligence. As to the reason for admission, we used three classes that may represent different types of patients: observation, diagnostics and therapy. We used SES on neighbourhood level with a score that is composed of three elements: low/high income, educational level and percentage of unemployed. We also used being a non-western immigrant as a case-mix variable. Immigrants are defined as those born outside the Netherlands or with at least one parent born outside the Netherlands. Finally, we added a hospital and diagnosis specific variable describing the percentage of day care admissions, to be sure that differences in LOS were not confounded by the policy of the hospital to treat patients on an in-patient basis or in day care.

### Analysis

For each combination of diagnosis/procedure and registration year, we fitted a linear mixed effects model to the log-transformed LOS to answer the main research question on between-hospital and within-hospital variation. The distribution of LOS of individual patients is skewed to the right. The log-transformation makes the dependent variable approximately normally distributed [32]. We corrected for case-mix by adding the aforementioned variables to the fixed part of the model. We applied indirect standardisation, i.e. the modelled outcomes were applied to the population at hand. Likelihood ratio tests (LRT) were performed to test the effect of all case-mix variables together on the outcome variable LOS. A random intercept for each hospital was included in the model to split total variance into within-hospital variance and between-hospital variance. For each registration year, we calculated total variance, the intraclass correlation coefficients (ICC) for hospitals and the coefficient of variation (CV) as absolute and relative measures of variability. The ICC represents the ratio of between-hospital variance and total variance.

The additional research questions of the study are related to the performance of individual hospitals in terms of LOS. In the regression models per registration year, the random intercepts for the individual hospitals reflected the variation between hospitals that cannot be attributed to the case-mix variables. Therefore, they can be used as a measure of hospitals’ performance irrespective of their patient population. The linear mixed models also yielded empirical Bayes estimates for the random intercepts, including their standard errors. The correlations between the ranks of the yearly estimates of the random effects were calculated. These correlations indicated whether hospitals’ performances were persistent over the years. Additionally, the correlations of the empirical Bayes estimates between the diagnoses and procedures were calculated to investigate whether individual hospitals showed similar performance across diagnoses or procedures.

The regression models were applied to the log LOS scale. In order to interpret the outcomes on the original LOS scale, the results were back transformed, taking into account that the estimates on the original LOS scale depend on both the estimated mean and variance on the log LOS scale (for more details see Additional file 1).

Data management and analyses were performed using SPSS Version 20 and R 2.10 [33].

## Results

Tables 2 and 3 show the mean, median and interquartile range (IQR) of the observed and log-transformed LOS. The median observed LOS and log-transformed LOS declined in all cases, between 67 % (CHOL) and 36 % (CHF and PNEU). Also, the IQR for the observed LOS decreased, whereas for the log-transformed LOS the same quantity showed more stable behaviour over time.

**Table 2:** Observed LOS for 1995, 2000, 2005 and 2010 (median/IQR) and number of admissions in the dataset for 1995 and 2010

	LOS (mean/median/IQR)				# admissions in dataset	
	1995	2000	2005	2010	1995	2010
AMIplus	10.1/9/7	9.2/8/7	7.6/6/6	6.1/4/6	28216	25619
CHOL	8.7/6/5	6.3/4/4	4.6/3/3	3.6/2/1	10976	12703
CVA	21/14/22	21.4/12/20	12/9/11	9.5/7/8	15124	20282
FEMUR	21.9/17/15	22.5/15/17	14.8/11/11	10.6/8/7	9033	11609
HIP	17.3/15/6	14/12/6	8.9/7/3	6.9/6/2	9415	13497
CHF	14/11/10	13/9/10	11.1/9/9	9.3/7/8	15348	17491
KNEE	18.9/17/7	14/12/6	8.8/8/4	6.6/6/2	3038	10264
PNEU	13.9/11/10	13.4/10/9	10.9/8/8	9.3/7/6	10249	20783
RESCOL	22.5/17/13	21.1/14/12	17.8/12/11	15.6/10/11	5005	5988

**Table 3:** Log-transformed LOS for 1995, 2000, 2005 and 2010 (mean/median/IQR)

	1995	2000	2005	2010
AMIplus	2/2.2/0.9	1.9/2.1/1	1.6/1.8/1.1	1.4/1.4/1.4
CHOL	1.9/1.8/0.8	1.6/1.4/0.8	1.2/1.1/0.9	1/0.7/0.4
CVA	2.5/2.6/1.5	2.4/2.5/1.6	2.1/2.2/1.3	1.9/1.9/1.1
FEMUR	2.8/2.8/0.9	2.7/2.7/1.1	2.4/2.4/0.9	2.1/2.1/0.8
HIP	2.8/2.7/0.4	2.5/2.5/0.5	2.1/1.9/0.4	1.8/1.8/0.3
CHF	2.3/2.4/0.9	2.2/2.2/1	2.1/2.2/1	1.9/1.9/1.1
KNEE	2.9/2.8/0.4	2.6/2.5/0.5	2.1/2.1/0.5	1.8/1.8/0.3
PNEU	2.3/2.4/0.9	2.2/2.3/0.9	2.1/2.1/1	1.9/1.9/0.8
RESCOL	2.9/2.8/0.7	2.8/2.6/0.7	2.6/2.5/0.8	2.5/2.3/0.9

Total variance on the log-scale (not shown) increased over time for AMIplus, CHF and RESCOL. For CVA, FEMUR, HIP and KNEE it was stable or increased up to 2001 and decreased thereafter. For CHOL and PNEU total variance showed a different pattern with a peak in 2006 and 2001 respectively. The magnitude of the total variance differed between disease groups, with high variance for CVA, AMIplus and CHF in almost all years and low variance for HIP and KNEE.

**Fig. 1:**
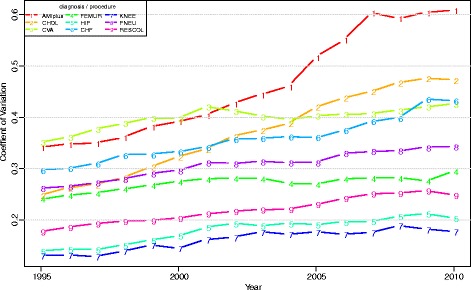
Coefficient of Variation of log-transformed LOS per diagnosis/procedure, between 1995 and 2010 (case-mix corrected on yearly basis)

Figure 1 shows the coefficient of variation (CV) of the log-transformed LOS. CV is a measure of relative variation (ratio of standard deviation and mean). Up to 2001, the CV increased for all diagnoses and procedures, whilst after 2001 the CV was stable for CVA, FEMUR, HIP and KNEE. Thus, even though total variance decreased for the latter four groups after 2001, the CV remained stable because of a steep decline of their mean LOS. For the other five diagnoses and procedures the CV continued to increase. The increase of the CV was largest for AMIplus and CHOL. The latter is caused by a large decrease of the mean LOS for both diseases, while for AMIplus a substantial increase in total variance also affected the increase of the CV.

**Fig. 2:**
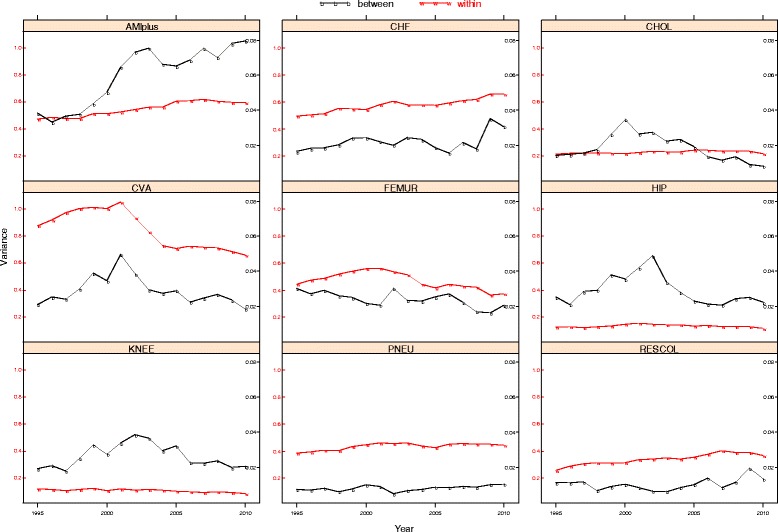
Between-hospital and within-hospital variances per diagnosis/procedure, between 1995 and 2010 (case-mix corrected on yearly basis)

Figure 2 shows the between-hospital and within-hospital variance for all diagnoses. The figure demonstrates that the magnitude of the between-hospital variance (right y-axis) is small compared to the within-hospital variance (left y-axis). In other words, the variation in log-LOS is largely explained by variation between individual patients within hospitals, even after case-mix adjustment, and only to a limited extent by variation between hospitals. Between-hospital variation was largest for KNEE and HIP (ICC between 0.15 and 0.25), smaller for AMIplus and CHOL (ICC between 0.05 and 0.15) and smallest for the other five disease groups (<0.05).

Figure 2 also demonstrates that for each diagnosis between-hospital and within-hospital variances generally follow similar trends. The variation between hospitals decreased from around 2001 onwards for CHOL, FEMUR, CVA, HIP and KNEE. The between-hospital variance increased for AMIplus, CHF, PNEU and RESCOL.

**Fig. 3:**
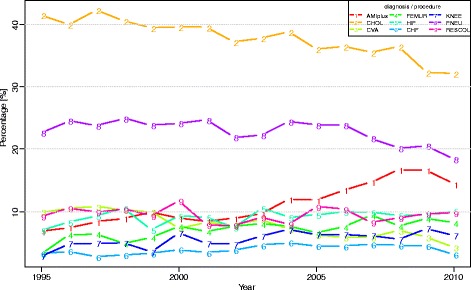
Percentage of variance of LOS explained by case-mix per diagnosis/procedure, between 1995 and 2010

The regression results (not shown here) demonstrated that age and the Charlson index had a significant impact on LOS across all diseases and years. The impact of gender and reason for admission was significant in all years for five out of nine diagnoses/procedures. The role of the other case-mix variables (neighbourhood SES, ethnicity and the percentage day-admissions (hospital level) was much more diffuse across diseases and years. In total, the case-mix variables explained between 0 and 10 % of the LOS variance for six diagnoses (Fig. 3). For AMIplus, this percentage was higher (10–20 %) between 2004 and 2010, while the role of case-mix variables was larger particularly for PNEU (20–25 %) and CHOL (30–40 %). Regarding CHOL, this higher percentage was caused by the variable type of treatment (open versus laparoscopic).

**Fig. 4:**
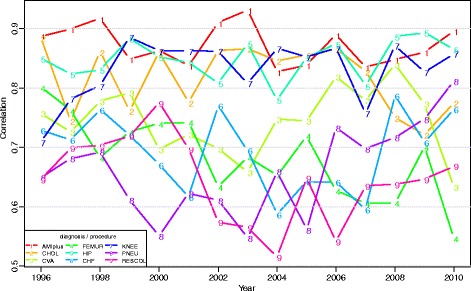
Correlation of hospital random effects with previous year per diagnosis/procedure, between 1995 and 2010

The analysis of the ranking of hospitals showed substantial correlation in hospitals’ performance between year t and year t + 1. Figure 4 shows a correlation of between 0.6 (PNEU) and 0.9 (AMIplus) for 1995 and 1996, for example. The figure also shows a different pattern across diseases. The correlation coefficients of the random effects showed a particularly strong correlation in hospital performance over time for AMIplus, CHOL, KNEE and HIP (correlation coefficient of between 0.7 and 0.9 across years). The correlation was much smaller for all other disease groups. Furthermore, there was little correlation (correlation coefficient <0.5) between hospitals’ performance (i.e. the hospital random effects) for separate diagnoses and procedures, except for HIP and KNEE (correlation coefficient of 0.8).

**Fig. 5:**
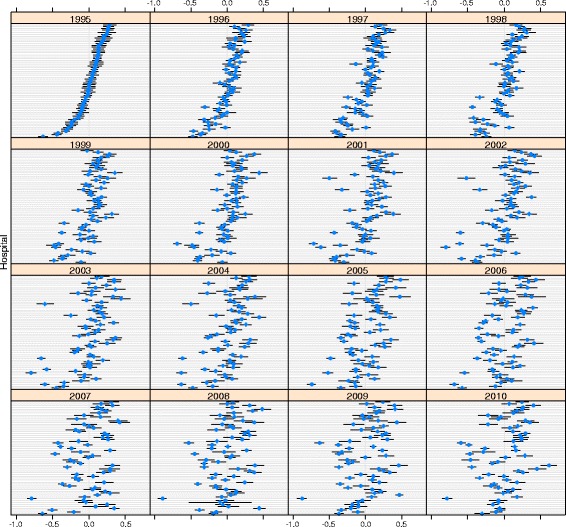
Relative hospital performance for AMIplus between 1995 and 2010

**Fig. 6:**
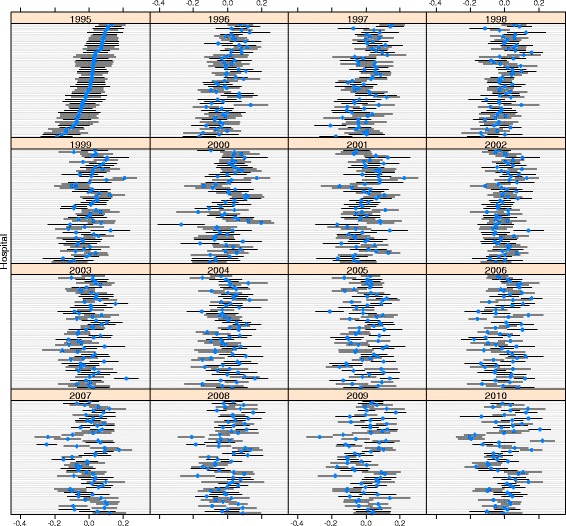
Relative hospital performance for PNEU between 1995 and 2010

Figures 5 and 6 illustrate the extent to which hospital rankings reflect significant differences between hospitals in terms of LOS. A diagnosis with high correlation in hospital performance over time (AMIplus), and one with low correlation (PNEU) are shown (the order of the hospitals on the y-axis is the same in all years). Fig. 6 shows that good and bad performers can be distinguished for AMIplus in 1995, even though some confidence intervals overlap. Several hospitals with average performance in 1995 had become good or bad performers in 2010. For PNEU the confidence intervals are much wider and hospitals can be distinguished to a lesser extent.

## Discussion

In this study, we investigated the variation in length of stay within and between Dutch hospitals over time. We adopted a diagnosis and procedure oriented approach. To our best knowledge, this study is the first to focus on these trends. In contrast to most previous studies, we analysed variation between (and within) hospitals instead of regions. We deemed this perspective more relevant, because decision making in hospital care largely takes place at the hospital level instead of the regional level. Furthermore, we included a broad set of diagnoses and procedures in order to provide a comprehensive analysis.

We found substantial differences between diagnoses and procedures in LOS variability and in temporal changes of LOS variance, demonstrating the relevance of a diagnosis and procedure specific approach. Between-hospital variance of standardised log-LOS appears to be small, both in absolute numbers and relative to total variance (ICC). Consequently, within-hospital variance roughly follows total variance. The relative variance (CV) did not decrease for any of the diagnoses/procedures on the log scale. Between-hospital variance generally followed the same pattern as within-hospital variance. Over time, hospital performance in terms of LOS was most stable for AMIplus, CHOL, HIP and KNEE (correlation >0.7). Regarding hospitals’ performance for separate diagnoses/procedures there was only substantial correlation between the two types of joint replacements. Finally, though between-hospital variance was significant overall, but in most cases pairwise differences between hospitals were not significant.

### Coefficient of variation

We expected the CV to increase less steeply from around 2000, because of the increasing use of instruments like clinical pathways and related fast track programs. The CV indeed ceased to increase for HIP, KNEE, CVA and FEMUR, despite a strong decrease of the ALOS. We surmise this is a result of establishing pathways across providers (hospitals, rehabilitation centres, nursing homes and home care), especially for the latter two diagnoses. For all other diagnoses, relative variation increased, however. AMIplus showed a remarkable CV pattern with a very strong increase up to 2007. This pattern can be explained by a downward shift in the LOS distribution leading to a lower ALOS, while the variance remained stable. This shift was not found for other diagnoses (see the histograms in Additional file 1).

These trends also indicate that the abolishment of budget caps for hospitals in 2001, in combination with unchanged or declining bed capacities, stimulated streamlining of care processes. No signs were found that the introduction of a DRG based reimbursement system in 2005 with freely negotiable prices affected LOS and variation in the following years.

### Between-hospital variation

The results showed that the variation in LOS was largely explained by variation between individual patients with variation between hospitals playing a minor role. It may be expected that the introduction of an innovation results in frontrunner hospitals and laggards and hence an increase of between-hospital variance for some years. This effect may have been attenuated, because we controlled for type of procedure, like minimal invasive versus open surgical procedure, and hence for related innovations.

Besides, the introduction of innovations that reduce LOS may reduce within-hospital variance (standardisation of processes) and increase between-hospital variance (front-runners and laggards). For colon resection, fast track procedures were introduced in the Netherlands in phases, with breakthrough programmes since 2006 [34]. For the development of between-hospital variance we found a zigzag pattern. However, the variance is very small, so changes may be accidental and may not be attributable to the introduction of fast track programmes. Fast track programmes for joint replacements were gradually introduced in Dutch hospitals from the beginning of this century. For these procedures, we found a shift from increasing to decreasing between-hospital variance in 2002. Between-hospital variance follows more or less the same pattern as the within-hospital variance, which can be attributed to the introduction of fast track programs in a growing number of hospitals. Remarkable is the increase of between-hospital variance for AMIplus throughout the period observed. Background of this finding might be an increase of inter-hospital transfers from hospitals without to hospitals with possibilities for primary invasive intervention (primary PCI), in line with guidelines, as this could result in better outcomes for patients. For CHF and for PNEU no clear change occurred in the period observed.

### Hospital ranking

There was no clear trend in hospital rank (based on the random effects) for separate diagnoses and procedures, which remained more or less constant over time. Hospitals performing well in year t mostly performed well in year t + 1 as well. This may be interpreted as supporting the use of LOS as performance indicator. Substantial changes in ranking would have thrown doubt on the reliability of LOS as an indicator. At the same time, pairwise differences between hospitals were not always significant. We found overlapping confidence intervals for hospital performance indicating that one should be careful when comparing individual hospitals. Except for HIP and KNEE, procedures of the same specialty (hospital department), there was no strong correlation between diagnoses/procedures in the performance of a hospital, indicating the absence of hospital wide policies.

### The influence of explanatory factors

The extent to which patient characteristics and type of medical intervention (together: case-mix variables) explained the variance in LOS, differed from year to year and between selected diagnoses and procedures. As to the impact of socio-economic status on LOS, this may not be surprising given the mixed findings of previous studies regarding this relation ([7, 22, 35–37]). For most diagnoses and procedures, the overall impact of case-mix was rather small. Also, we found that type of hospital had a very small but statistically significant effect on LOS. Previous studies found that the variation between doctors within hospitals was significantly smaller than the variation between hospitals, which we found to be small itself, or even insignificant [21, 38]. Therefore, only a small part of the variation in LOS between patients seems attributable to supply factors. A substantial part of the LOS variation remained unexplained.

### Is LOS approaching the bottom?

The results of our analysis led to the further question: is LOS approaching the bottom? In order to assess this, we performed additional analyses of the CV on the original LOS scale. For that purpose, we estimated a separate linear mixed effects model on the pooled data including a time-trend (for more details see Additional file 1).

**Fig. 7:**
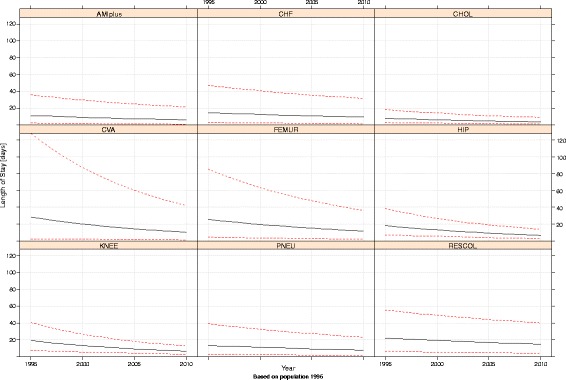
Modelled LOS plus 95% confidence interval per diagnosis/procedure, between 1995 and 2010

**Fig. 8:**
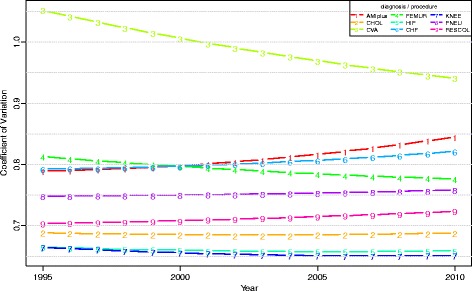
Modelled coefficient of variation of LOS per diagnosis/procedure, between 1995 and 2010 (case-mix corrected using direct standardisation to the 1995 population)

Figure 7 shows for each diagnosis/procedure, after back-transformation, the longitudinally modelled LOS as well as its confidence interval over time. As can be seen, the confidence bands narrow more rapidly for CVA, FEMUR, HIP and KNEE, in accordance with the decreasing CV’s. These results made it possible to model the CV on the original LOS scale (Fig. 8). On that scale, CV decreased for four out of nine diagnoses. In the observed period, this decrease did not tend to flatten off, not even for HIP and KNEE with their comparatively low variance. These trends suggest that LOS has not yet reached the bottom. The same conclusion can be drawn for AMIplus, CHF, PNEU and RESCOL, which show a comparatively high variation, while their CV increased or was stable in the observed period. For CHOL, CV was low and stable throughout the observed period, suggesting little room for improvement.

There can be a point below which a shorter LOS results in negative effects, e.g. in terms of quality, though currently there is little empirical evidence that this point is being reached [8, 39]. Furthermore, future improvements in medical technology (for both hospital and follow-up care) may provide opportunities for further LOS reductions without such negative effects. As to higher readmission rates, which could be an unwanted consequence of too short LOS, several studies have shown that a relative short LOS need not be associated with higher readmission rates [40–43]. This has been confirmed in more recent studies for AMI [44] and colon resection [14, 45–47]. For the Netherlands, this has not yet been investigated systematically.

### Implications for policy and practice

In general, our results suggest that efficiency improvement policies in hospitals should focus on specific patient groups (diagnoses) or procedures, since hospitals may now perform well in one area but worse in others. For most diagnoses and procedures, it appears possible to further reduce the (variation in) LOS. This implies that (certain) hospitals may be able to reduce the treatment cost per inpatient stay and to treat a greater number of patients, given their capacity and financial resources. Otherwise, hospitals could treat the same number of patients and reduce their capacity. The latter may be preferable from a societal perspective, because leaving the capacity unchanged may tempt hospitals and physicians to generate demand e.g. by lowering thresholds for admission (supplier induced demand [9]). This could create an unnecessary increase in health care spending.

If further reductions in the variation in LOS are possible, the question arises how to realize them? Hospitals could create and/or use benchmarking data in order to compare their performance with other hospitals and try to learn from each other using in-depth data analyses and/or discussions. Health care purchasers may also use this type of information in negotiations with hospitals in order to try to stimulate efficiency in health care delivery. However, between-hospital comparisons should be made with great care, including comprehensive case-mix adjustment and taking into account statistical uncertainty (e.g. by presenting confidence intervals) [48].

Finally, with further LOS reductions, it is likely that the need for care outside the hospital will increase. Therefore, establishing pathways with better coordination across providers in the care chain is required.

### Limitation and follow up research

The results should be interpreted with the following limitations in mind. For technical reasons, individual level data on household income from the national register could be linked to admission data from 2003 onwards only. We performed additional sensitivity analyses with individual-level household income as extra case-mix variable for this subset of years. It appeared that the effect of this individual SES variable was rather diffuse over time and its effect on LOS was negligible in all cases. Second, due to insufficient data, we could not include the educational level of patients. Third, it is possible that part of the variance was due to remaining heterogeneity between patients with a certain diagnosis. The adjustment for comorbidity is a complex issue, since many types of comorbidities exist and their presence may vary between patient groups. In this study, we used the Charlson index which covers a wide set of comorbidities, but is likely not to provide perfect case-mix adjustment. With regard to the statistical analysis, future studies may try to apply more flexible distributions for the modelling of LOS, such as the (generalized) Gamma or Weibull distribution [32]. Finally, future studies could take a broader perspective, covering the entire care pathway including health care outside the hospital. This would give insight into whether shorter LOS is compensated with greater health care use outside the hospital.

## Conclusion

In conclusion, we argue that detailed trend analyses of LOS present useful figures for a better understanding of the (impact of) changes in hospital care. We found clear differences between diagnoses and absence of hospital-wide policy. LOS is not systematically reaching a bottom, so further efficiency improvements seem possible. Finally, policymakers and health care purchasers should take into account statistical uncertainty when comparing LOS between hospitals and identifying inefficient hospitals.

## Additional file

Additional file 1:
**Appendix I.** Search strategy. **Appendix II.** Descriptives. **Appendix III.** Details trend analysis and back transformation. (DOCX 38 kb)

## Footnotes

1 Fast track is a program of pre-, peri- and post-operative interventions that enhances convalescence and shortens LOS [11, 12] without significant effects on complications and readmissions [13]. Clinical pathways (CP’s), also named integrated pathways, critical pathways, care pathways or care maps, are structured multidisciplinary care plans, that detail essential steps in the care of patients with specific medical problems [14]. They aim to link evidence to practice and optimise clinical outcomes whilst maximising clinical efficiency [15].

2 See Additional file 1 for search strategy.

3 Statistics Netherlands, http://statline.cbs.nl/statweb/

4 Unlike other countries, the Dutch system covers the entire treatment episode also enclosing outpatient care. From 2005 until 2012 the system was very detailed including 30,000 different groups.

5 http://www.scp.nl/english/

6 http://www.depraktijkindex.nl/
