# Supplementary material for: Has variation in length of stay in acute hospitals decreased? Analysing trends in the variation in LOS between and within Dutch hospitals
Source: BMC Health Serv Res. 2015 Sep 30;15:438. doi: 10.1186/s12913-015-1087-6 (PMC4590267; doi:10.1186/s12913-015-1087-6)
Supplement: Additional file 1: — Appendix I. Search strategy. Appendix II. Descriptives. Appendix III. Details trend analysis and back transformation. (DOCX 38 kb) [file 12913_2015_1087_MOESM1_ESM.docx]

**APPENDICES**

Appendix I – Search strategy

Database: MEDLINE 1990 to first half of 2013, MEDLINE In-Process & Other Non-Indexed Citations

Search Strategy:

--------------------------------------------------------------------------------

1 (stay length* or stay variation* or (duration adj4 stay*) or (length adj4 stay*) or (duration adj4 hospitali?ation*) or (length adj4 hospitali?ation*) or (los and stay*)).tw. (41266)

2 "length of stay"/ (49320)

3 1 or 2 (72224)

4 (nursing homes/ or residential facilities/) not (hospitals/ or hospitalization/) (28679)

5 3 not 4 (71576)

6 variation*.tw. (393983)

7 5 and 6 (1664)

8 (time trends or time factors or period or periods or decade*).tw. (1068955)

9 (19??-19?? or 19??-20?? or 20??-20??).tw. (216502)

10 time factors/ (920334)

11 7 and (8 or 9 or 10) (552)

12 limit 11 to yr=1990-2011 (506)

13 remove duplicates from 12 (483)

Appendix II – Descriptives

*Histograms of log-transformed LOS for AMIplus and FEMUR*

*Descriptives (median and inter quartile range (IQR)) for continuous case-mix variables: Neighbourhood SES, Charlson index, % day care per hospital for the registration year 2010*

|  | Neigbourhood SES | | Charlson Index | | % day care (per hosp.) | |
| --- | --- | --- | --- | --- | --- | --- |
| diagnosis | median | IQR | median | IQR | median | IQR |
| AMIplus | 0.19 | 1.21 | 0.00 | 1.00 | 1.25 | 4.10 |
| CHOL | 0.09 | 1.21 | 0.00 | 0.00 | 0.54 | 5.73 |
| CVA | 0.17 | 1.20 | 0.00 | 1.91 | 3.88 | 6.68 |
| FEMUR | 0.13 | 1.24 | 0.00 | 0.00 | 0.00 | 0.60 |
| HIP | 0.04 | 1.16 | 0.00 | 0.00 | 0.00 | 0.00 |
| CHF | 0.23 | 1.26 | 0.00 | 3.76 | 2.09 | 3.79 |
| KNEE | 0.11 | 1.21 | 0.00 | 0.00 | 1.52 | 2.23 |
| PNEU | 0.15 | 1.28 | 0.00 | 2.54 | 1.74 | 3.69 |
| RESCOL | 0.07 | 1.20 | 0.00 | 8.05 | 0.00 | 0.00 |

*Descriptives for case-mix variable sex for 2010 (%)*

| diagnosis | | male | | female | |
| --- | --- | --- | --- | --- | --- |
| AMIplus | | 33.9 | | 66.1 | |
| CHOL | | 68.5 | | 31.5 | |
| CVA | | 49.3 | | 50.7 | |
| FEMUR | | 69.3 | | 30.7 | |
| HIP | | 68 | | 32 | |
| CHF | | 50.3 | | 49.7 | |
| KNEE | | 67.7 | | 32.3 | |
| PNEU | | 42.9 | | 57.1 | |
| RESCOL | | 51 | | 49 | |

*Descriptives for case-mix variable age class for 2010 (%)*

| diagnosis | [0-25) | [25-40) | [40-55) | [55-70) | [70-85) | [85-Inf] |
| --- | --- | --- | --- | --- | --- | --- |
| AMIplus | 0.1 | 1.7 | 17.1 | 36 | 36.2 | 9 |
| CHOL | 3.7 | 19.2 | 32.1 | 28.6 | 14.9 | 1.5 |
| CVA | 0.8 | 1.6 | 11.2 | 27.2 | 43.9 | 15.3 |
| FEMUR | 0.7 | 1 | 4.5 | 14.6 | 42.9 | 36.2 |
| HIP | 0.1 | 0.8 | 7.4 | 39.2 | 47.4 | 5 |
| CHF | 0.1 | 0.6 | 3.7 | 17.6 | 49.5 | 28.4 |
| KNEE | 0 | 0.2 | 7.5 | 43.2 | 45.6 | 3.4 |
| PNEU | 12.6 | 4.2 | 9.7 | 22.5 | 36.8 | 14.2 |
| RESCOL | 1.9 | 3.9 | 12.5 | 32.9 | 40.7 | 8.1 |

*Descriptives for case-mix variable admission reason for 2010 (%)*

| diagnosis | diagnostic | observational | therapeutic |
| --- | --- | --- | --- |
| AMIplus | 7.1 | 6.2 | 86.7 |
| CHOL | 1.1 | 0.6 | 98.3 |
| CVA | 10 | 9.3 | 80.7 |
| FEMUR | 1.5 | 1.1 | 97.4 |
| HIP | 0.8 | 0.1 | 99.2 |
| CHF | 5.5 | 7.5 | 87.1 |
| KNEE | 0.6 | 0.1 | 99.3 |
| PNEU | 5 | 8.3 | 86.7 |
| RESCOL | 3.2 | 1 | 95.8 |

Appendix III – Details trend analysis and back transformation

In order to model a possible time trend we fitted for each diagnosis a linear mixed effects model to the log transformed LOS. Registration year was included in the fixed effects part and random intercepts and slopes for the individual hospitals were added to describe the between-hospital variance. Additionally, the within-hospital variance was modelled by an exponential function over time. Using these models, direct standardisation to the patient population of 1995 was performed.

Fitting a linear (mixed) model to the log transformed LOS observations, implies that the LOS observations itself follow a lognormal distribution. The mean and variance of this distribution can be expressed as functions of the mean and variance of the log-transformed observations.: ${mean= e}^{\mu+\sigma^{2}/2}$, ${var =\left( e^{\sigma^{2}}-1 \right)\cdot e}^{2\mu+\sigma^{2}}$, where µ and σ^2^ are the mean and variance of the logarithmic transformed-LOS. Thus, for inference on the original LOS scale we have to use these formulas to back transform the results from the linear regression models on the log LOS observations.
